# Optimization of cell viability assays to improve replicability and reproducibility of cancer drug sensitivity screens

**DOI:** 10.1038/s41598-020-62848-5

**Published:** 2020-04-02

**Authors:** Peter Larsson, Hanna Engqvist, Jana Biermann, Elisabeth Werner Rönnerman, Eva Forssell-Aronsson, Anikó Kovács, Per Karlsson, Khalil Helou, Toshima Z. Parris

**Affiliations:** 1 Department of Oncology, Institute of Clinical Sciences, Sahlgrenska Cancer Center, Sahlgrenska Academy at University of Gothenburg, Gothenburg, Sweden; 2 Department of Clinical Pathology, Sahlgrenska University Hospital, Gothenburg, Sweden; 3 Department of Radiation Physics, Institute of Clinical Sciences, Sahlgrenska Cancer Center, Sahlgrenska Academy at University of Gothenburg, Gothenburg, Sweden

**Keywords:** Cancer, Drug discovery, Molecular biology, Oncology

## Abstract

Cancer drug development has been riddled with high attrition rates, in part, due to poor reproducibility of preclinical models for drug discovery. Poor experimental design and lack of scientific transparency may cause experimental biases that in turn affect data quality, robustness and reproducibility. Here, we pinpoint sources of experimental variability in conventional 2D cell-based cancer drug screens to determine the effect of confounders on cell viability for MCF7 and HCC38 breast cancer cell lines treated with platinum agents (cisplatin and carboplatin) and a proteasome inhibitor (bortezomib). Variance component analysis demonstrated that variations in cell viability were primarily associated with the choice of pharmaceutical drug and cell line, and less likely to be due to the type of growth medium or assay incubation time. Furthermore, careful consideration should be given to different methods of storing diluted pharmaceutical drugs and use of DMSO controls due to the potential risk of evaporation and the subsequent effect on dose-response curves. Optimization of experimental parameters not only improved data quality substantially but also resulted in reproducible results for bortezomib- and cisplatin-treated HCC38, MCF7, MCF-10A, and MDA-MB-436 cells. Taken together, these findings indicate that replicability (the same analyst re-performs the same experiment multiple times) and reproducibility (different analysts perform the same experiment using different experimental conditions) for cell-based drug screens can be improved by identifying potential confounders and subsequent optimization of experimental parameters for each cell line.

## Introduction

Cancer drug candidates currently have the lowest overall success rates and are 23% less likely to succeed in phase III clinical trials compared with other therapeutic areas^1–3^. At a cost of approximately $3 billion per approved drug, over a decade may have passed from target discovery to drug approval (long development time) and tens of thousands of drug candidates would have likely been dropped due to drug safety and/or efficacy issues^4^. Cell-based pharmacogenomics screens are commonly used during the preclinical drug screening process to identify druggable targets by characterizing the biological effects associated with drug response and toxicity. However, low interlaboratory reproducibility of cell-based pharmacogenomics screens, lack of robust disease models that recapitulate the natural progression of human cancers, and drug-associated toxicity issues contribute to the high drug attrition rates in oncology^5–13^. Biomedical researchers and the pharmaceutical industry are, therefore, developing strategies to improve early-phase drug screening, *e.g*. novel preclinical models of disease and drug repurposing^9,14^.

Drug-dose response assays (*e.g*. MTT assay) performed in two-dimensional (2D) cell culture are typically used to evaluate drug efficacy and potency in cells exposed to a drug for up to 72 hours^15–17^. However, it has been challenging to develop robust drug sensitivity assays that produce consistent results within a single lab and/or across multiple laboratories because similarly designed drug screening studies have shown varying results^7,16^. Although several recent studies have attempted to address this issue by describing biological (*e.g*. differences in cell type, medium composition, seeding density) and technical factors (*e.g*. edge effect, as well as, differences in assay, drug concentration and treatment time, methods for cell counting) contributing to data replicability (the same analyst re-performs the same experiment multiple times) and data reproducibility (different analysts perform the same experiment using different experimental conditions, *e.g*. cell culture systems and reagents), few studies have proposed a strategy to identify and correct for sources of variability in drug response^6,7,16,18,19^. Regardless of the method used to determine cellular response to drug treatment or whether automated or manual lab work is employed, assay robustness testing is recommended. Ideally, a robust assay should be stable over replicate experiments and between labs. Therefore, minor variations in assay parameters (*e.g*. medium composition, seeding density, drug storage) should not drastically affect the results^19^.

Newly developed drug sensitivity assays should first be optimized to establish signal dynamic range using quality control metrics (QCM; *e.g*. Analysis of Variance (ANOVA), Z-factor (Z), Signal window (SW) and coefficient of variation (CV)) in single wells or on a plate level, followed by implementation of the optimized experimental parameters using a well-established drug and cell line^19–21^. Thereafter, the optimized assay should be validated and the results reported using suitable drug response metrics (*e.g*. IC50 (half maximal inhibitory concentration), Emax (maximum effect on viability at maximal drug concentration), AUC (area under the dose-response curve), GR50 (growth rate inhibition concentration at GR(c) = 0.5), GRmax (maximal measured GR value), GR_AOC_ (area over the curve), DSS (drug sensitivity scoring), and NDR (normalized drug response)) to evaluate drug potency (GR50, IC50), efficiency (GRmax, Emax), and drug response (GR_AOC_, AUC, DSS, NDR). Due to differences in cellular division rates, metrics for growth inhibition (GR50, GRmax, GR_AOC_, and NDR) have been shown to produce more consistent interlaboratory results than conventional metrics (IC50, Emax, and AUC)^6,16,22–25^.

Here, we propose a strategy to identify potential confounders in a 2D *in vitro* drug response screening assay (*e.g*. cell culture conditions, drug storage, drug-dose response assay parameters, drug response metrics) that can affect data replicability, data reproducibility, and cell viability^26^. In addition, we show that suboptimal assay conditions can be improved, and together with suitable dose-response metrics can result in consistent data between laboratories.

## Results

### Replicability was affected by suboptimal resazurin reduction assay and cell culture protocols

**Figure 1:**
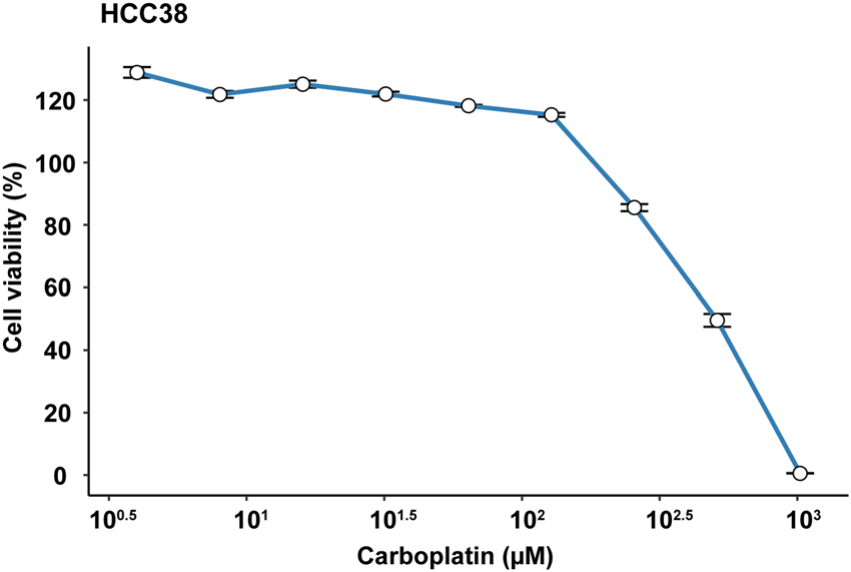
The effect of suboptimal experimental parameters on dose response estimates. HCC38 breast cancer cells were treated with 2–1024 µM carboplatin (for 24 hours) that had been stored at −20°C. The dose-response curve was generated using ggplot2 (version 3.2.1) in R^51^.

To be able to compare drug potency estimates between different studies, long-established breast cancer cell lines (HCC38 and MCF7) and pharmaceutical agents (bortezomib, cisplatin, and carboplatin) in cancer research and drug screening were utilized. To investigate the effect of these chemotherapeutic agents on cell viability in MCF7 and HCC38 cells, we initially performed the resazurin reduction assay using standard experimental protocols and calculated dose-response curves and the half-maximal inhibitory concentration (IC50) after 24-hour drug exposure. Cells were plated at a density of 1.0 × 10^4^ cells per 96-well in 100 µl HuMEC basal serum-free medium. Previous studies have shown that complete growth medium supplemented with FBS reduces the effect of proteasome inhibitor bortezomib on proteasome activity^27^, thereby warranting the use of HuMEC medium for all drug treatments. Due to poor aqueous solubility, the pharmaceutical drugs were dissolved in DMSO (final DMSO concentration ranging from 0.0002–10%) according to the manufacturer’s instructions, further diluted with 1xPBS to the desired working concentration (1–10000 nM bortezomib and 2–1024 µM cisplatin/carboplatin), and stored at 4 °C for up to one week in Falcon TC-treated flat-bottom culture microplates (VWR). However, use of these experimental parameters (cell density of 1.0 × 10^4^ per 96-well plate, serum-free medium, and a single DMSO control) resulted in intra- and interexperimental inconsistencies, including differences between replicates and dose-response curves with viability estimations higher than 100% at the start of the experiment (Fig. 1).

### Cell viability was negatively affected by evaporation and DMSO solvent

**Figure 2:**
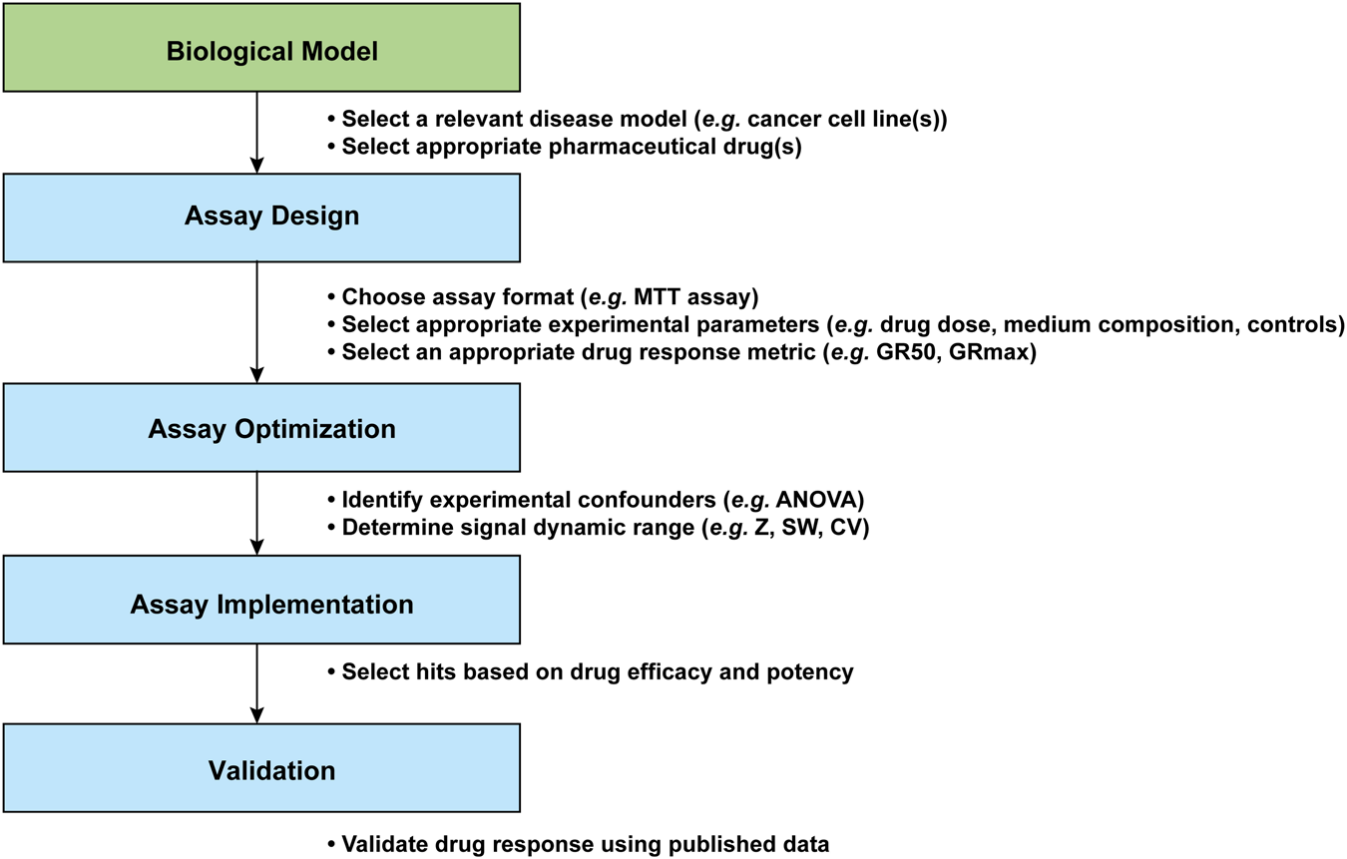
Schematic workflow of the experimental design. The resazurin viability assay was used to evaluate drug response using two breast cancer cell lines (MCF7 and HCC38) and three pharmaceutical drugs (bortezomib, carboplatin and cisplatin). The assay was optimized using Analysis of Variance (ANOVA) to identify potential experimental confounders and Z-factor (Z), Signal window (SW) and coefficient of variation (CV) to evaluate the signal dynamic range. Thereafter, the optimized parameters were implemented using four breast cancer cell lines (MCF7, HCC38, MCF-10A, and MDA-MB-436) and three pharmaceutical drugs (bortezomib, carboplatin and cisplatin). Finally, our data were compared to published data (pharmacoDB^42^ and Hafner *et al*.^31^).

**Figure 3:**
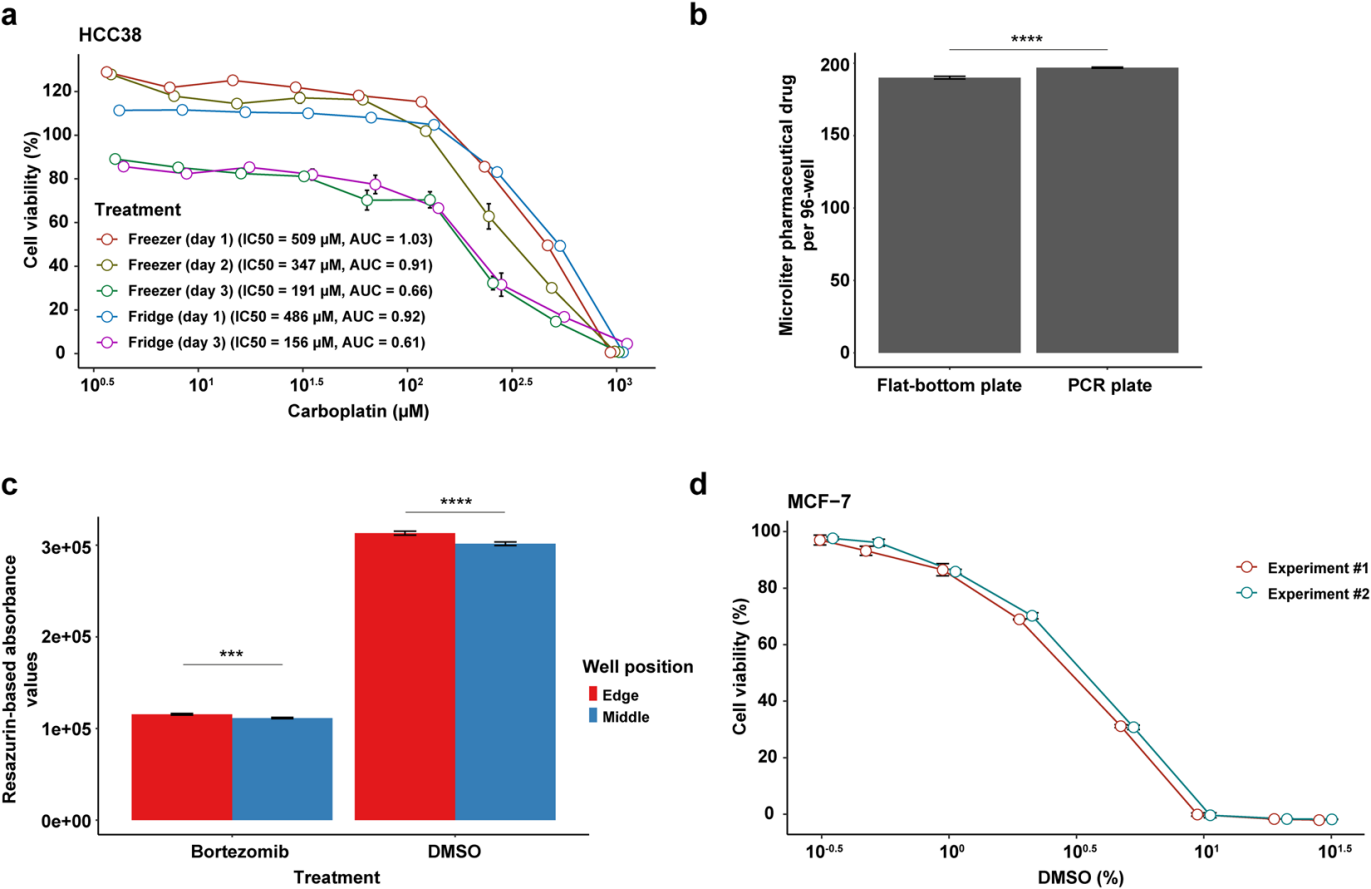
Impact of drug storage, evaporation, and DMSO concentration on cell viability in breast cancer cell lines. (**a**) HCC38 breast cancer cells were treated with 2–1024 µM carboplatin (for 24 hours) that had been stored at 4 °C (fridge) or −20 °C (freezer thawed daily). (**b**) Standard PCR plates (sealed with aluminum tape) were shown to result in less evaporation of pharmaceutical drugs during storage. Wilcoxon test was used to calculate statistical significance (Benjamini-Hochberg adjusted p-values). ns = not significant (P > 0.05); *P ≤ 0.05; **P ≤ 0.01; ***P ≤ 0.001; ****P ≤ 0.0001. (**c**) The perimeter wells of flat-bottom culture microplates exhibited higher resazurin-based absorbance values than wells in the middle of the plate, indicating less evaporation in the middle wells. Wilcoxon test was used to calculate statistical significance (Benjamini-Hochberg adjusted p-values). ns = not significant (P > 0.05); *P ≤ 0.05; **P ≤ 0.01; ***P ≤ 0.001; ****P ≤ 0.0001. (**d**) MCF7 breast cancer cells were treated with 0.33–10% (v/v) DMSO for 24 hours. Cell viability was determined using the resazurin reduction assay with 10% resazurin solution incubated for four hours. Error bars depict the standard error of the mean. The dose-response curves and bar plots were generated using ggplot2 (version 3.2.1) in R^51^.

To evaluate the effect suboptimal experimental design has on cell viability (IC50) and the area under the dose-response curve (AUC) values, we tested several experimental parameters that may affect replicability and reproducibility (*e.g*. storage of the pharmaceutical drugs, cell culture conditions, and resazurin assay conditions; Table 1 and Fig. 2). We first examined whether evaporation had a negative impact on cell viability and should, therefore, be minimized. Evaporation rates and/or cell viability were measured for a) diluted drugs stored at 4 °C or −20 °C for 48 hours in 96-well flat-bottom culture microplates sealed with Parafilm around the lid, b) diluted drugs stored at −20 °C for 72 hours in either PCR plates (sealed with aluminum tape) or culture microplates (sealed with Parafilm around the lid), and c) HCC38 cells treated with DMSO, PBS or diluted drugs incubated at 37 °C in a humidified 5% CO_2_ environment. After as little as 48 hours storage at 4 °C or −20 °C, evaporation and eventual concentration of the diluted drugs had a significant effect on cell viability (Fig. 3a). Consequently, IC50 and AUC values decreased with time, but storage of the drugs in the refrigerator or freezer (the same length of time) had no effect on cell viability. However, evaporation of the drugs occurred at a faster rate in flat-bottom culture microplates (Fig. 3b). Despite using culture microplates that are designed to minimize evaporation, an edge effect was observed in wells around the perimeter of microplates incubated at 37 °C (Supplementary Figure 1). Consequently, elevated resazurin-based absorbance values were measured for cells in the perimeter wells, with similar effects found for cells treated with DMSO and bortezomib (Fig. 3c).

**Table 1:** Assay optimization parameters.

Parameter	Conditions tested^*^	Duration of drug treatment	Optimal conditions
**Evaporation in 96-well microplates**			
*Drugs stored in flat-bottom culture microplates*	4 °C, −20 °C	48 h	Short-term storage (<7 days) at 4 °C or −20 °C
*Drugs stored in different 96-well microplates*	PCR, flat-bottom culture plates; −20 °C	48 h, 72 h	PCR plates with aluminum sealing tape
*Cells incubated in flat-bottom culture microplates in the incubator*	Bortezomib, DMSO	24 h, 48 h, 72 h	Avoid perimeter wells (rows A and H; columns 1 and 12) due to edge effects
*Edge effect in the incubator*	Bortezomib, DMSO, PBS	24 h	Avoid perimeter wells (rows A and H; columns 1 and 12) due to edge effects
**Cell density and drug sensitivity**			
*Seeding density*	5.0 × 10^3^, 7.5 × 10^3^ or 1.0 × 10^4^ cells per well	24 h	7.5 × 10^3^ cells per 96-well
*Sensitivity to drug solvent (DMSO)*	0.33, 0.5, 1, 2, 5, 10, 20, 30% (v/v) DMSO	24 h	<1% (v/v) DMSO
**Medium**			
*Medium type*	Growth medium + 0% FBS, 5% FBS, 10% FBS, 15% FBS or HuMEC serum-free medium	24 h	Growth medium + 10% FBS
*Medium volume*	100 µl, 200 µl, 240 µl growth medium + 10% FBS	24 h	100 µl
*Medium/drug renewal*	With, without medium/drug renewal every 24 h	24 h, 48 h, 72 h	Without medium/drug renewal
*Antibiotics*	With, without penicillin-streptomycin	24 h	With or without penicillin-streptomycin
**Controls**			
*DMSO controls*	Matched DMSO concentration controls, single DMSO control	24 h	Matched DMSO concentration controls
**Resazurin**			
*Mode of resorufin detection*	Absorbance, fluorescence	24 h	Absorbance or fluorescence
*Incubation time*	1 h, 2 h, 4 h, 6 h	24 h	≥4 h
*Concentration*	5%, 10%, 15%, 20% resazurin	24 h	10% resazurin
*Cross-reactivity with pharmaceutical compounds*	Growth medium + 10% FBS (without cells) incubated with bortezomib, carboplatin, or DMSO	24 h	No cross-reactivity observed

^*^Unless otherwise specified, the experiments were performed using cells seeded at a density of 7.5 × 10^3^ cells per well in 100 µl growth medium supplemented with 10% FBS, followed by drug treatment for 24 hours at 37 °C. Cells were then incubated with 10% resazurin solution for 4 hours.

We then evaluated experimental conditions (*e.g*. sensitivity to DMSO, medium type, resazurin incubation time) that could potentially have a detrimental effect on the performance of the resazurin assay or cell viability (Table 1). Major DMSO cytotoxic effects on MCF7 cells were observed after 24 hours exposure to as little as 1% (v/v) DMSO, with a substantial decrease in cell viability with increasing DMSO concentration (Fig. 3d). In addition, use of a single DMSO vehicle control containing 1% (v/v) DMSO resulted in dose-response curves starting at cell viability higher than 100% and relatively large error bars, which was corrected by using matched DMSO concentration controls for each drug dose. All other tested experimental conditions were frequently cell line-specific and had minimal effects on the dose-response curves. Nevertheless, stable dose-response curves with small error bars were produced using 7.5 × 10^3^ cells per 96-well in 100 µl growth medium containing 10% FBS, without daily renewal of the medium/drug or supplementing the growth medium with antibiotics. These culture conditions allowed the cells to be cultured for at least 72 hours without reaching the plateau-phase in growth, no detectable production of the non-fluorescent dihydroresorufin or affecting the proteasome inhibitory properties of bortezomib. Furthermore, both absorbance and fluorescence were comparable methods of resorufin detection and no cross-reactivity was observed between resazurin solution (10% (w/v) resazurin solution incubated for four hours) and the tested drugs in growth medium containing 10% FBS.

### Experimental parameters should be optimized due to the influence on cell viability

**Figure 4:**
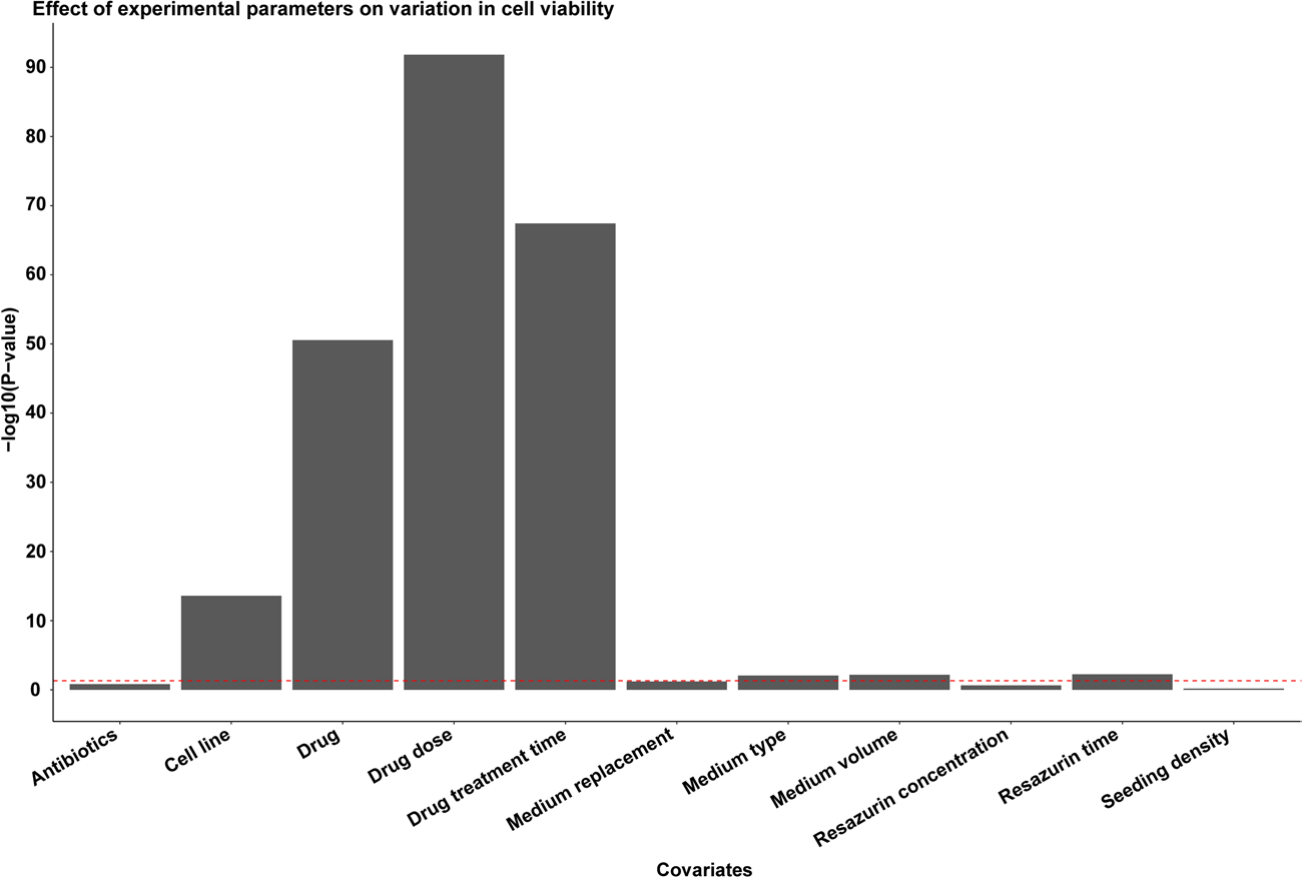
One-way ANOVA shows the influence of experimental parameters on cell viability during cancer drug screening. Cell viability was used as the dependent variable and all other experimental parameters as covariates. The red dotted line depicts the threshold for statistical significance at -log10(*P* = 0.05). The bar plot was generated using ggplot2 (version 3.2.1) in R^51^.

To identify factors influencing cell viability, one-way ANOVA was then employed using cell viability as the dependent variable and all other experimental parameters (*e.g*. cell line, drug, drug dose) as covariates. As expected, the vast majority (approximately 90%) of the variation in cell viability was associated with the choice of pharmaceutical drugs (*i.e*. drug type, dose, and treatment time) and cell line (5.4%; Supplementary Table 1). Though to a lesser extent, growth medium (type of medium/addition of FBS and medium volume) and resazurin incubation time also had an impact on cell viability (*P* < 0.05; Fig. 4). These findings highlight the importance of assay optimization for each cell line to minimize the effect of confounders.

### Optimized assay conditions facilitated reproducible cancer drug cytotoxicity testing

**Figure 5:**
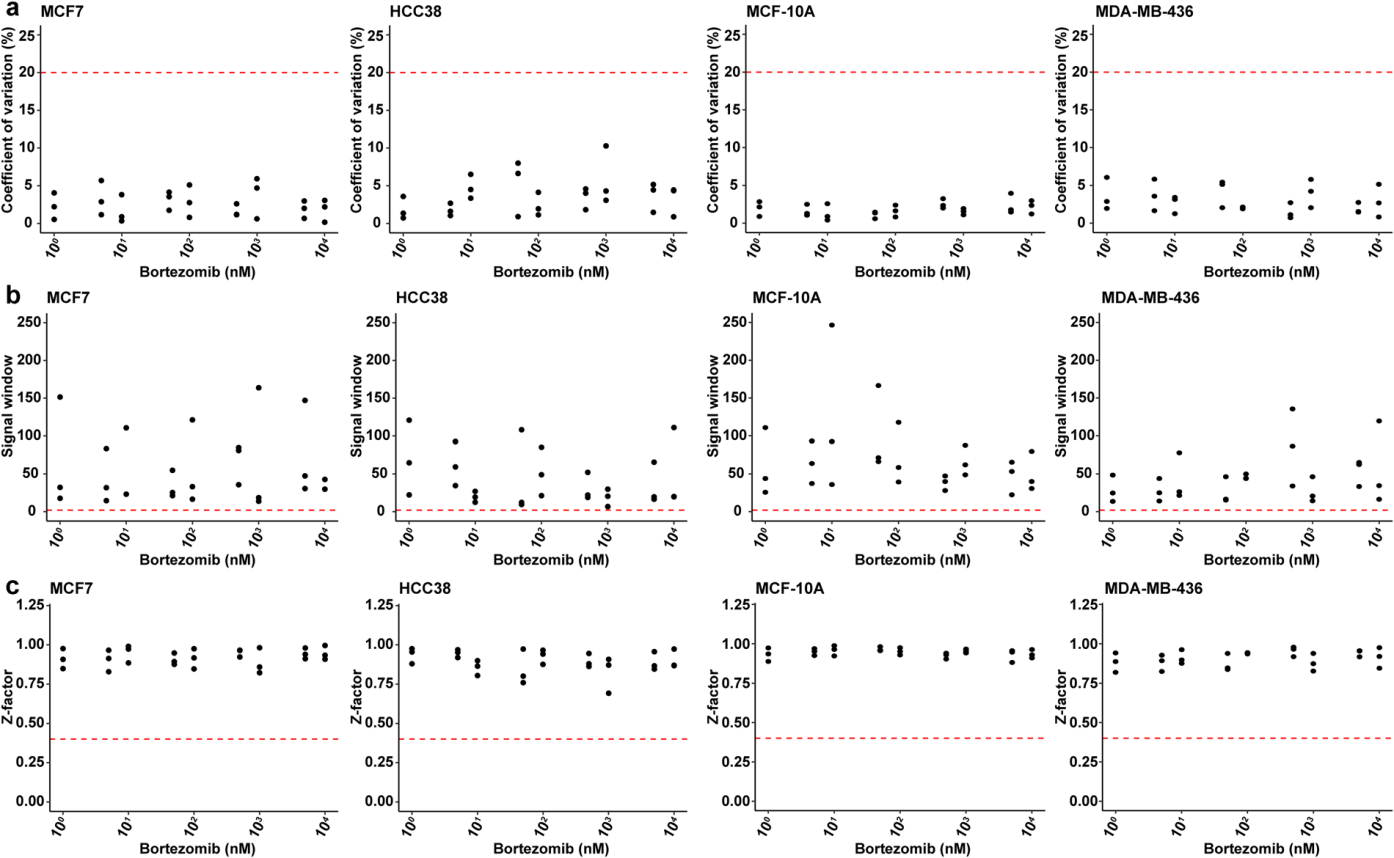
Validation of the optimized resazurin reduction assay using assay quality control metrics (coefficient of variation (CV), signal window (SW), and Z-factor (Z)). (**a**) CV, (**b**) SW, and (**c**) Z were determined using cell viability data for MCF7, HCC38, MCF-10A, and MDA-MB-436 breast cancer cells treated with bortezomib for 24 hours. Three independent experiments were performed in triplicate. The red dotted lines depict cutoffs set at CV < 20%, SW > 2, and Z > 0.4. The scatterplots were generated using ggplot2 (version 3.2.1) in R^51^.

To validate the optimized resazurin reduction assay for drug cytotoxicity testing, we determined IC50, GR50, and GRmax values for MCF7, HCC38, MCF-10A, and MDA-MB-436 cells exposed for 24 hours with bortezomib or cisplatin (cell seeding density of 7.5 × 10^3^ cells per 96-well in 100 µl growth medium supplemented with 10% FBS). All experiments were performed at least three times in triplicate. Intra- and interexperimental variation in resazurin-based absorbance values were also examined using assay QCM (Z-factor, signal window (SW), coefficient of variation (CV)) with thresholds set to Z-factor > 0.4, SW > 2, and CV < 20%. An ideal assay would have a large signal dynamic range and/or small signal variation (standard deviation (SD) or CV), with a Z-factor close to or equal to 1^28^. In addition, the signal window is recommended to be at least 2 SD the largest detected assay signal to differentiate biologically active and inactive compounds^29^. Here, we show that the optimized assay performed well in all four cell lines using bortezomib and cisplatin. All data points were within the recommended ranges, with the majority of data points for Z-factor > 0.75, SW > 10, and CV < 5% (Fig. 5 and Supplementary Table 2).

**Figure 6:**
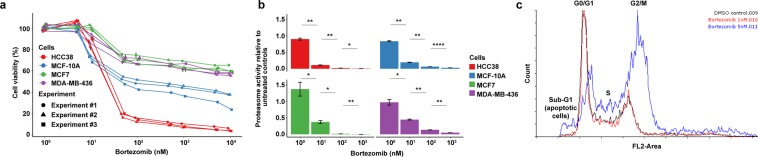
Cell viability data generated after optimization of the resazurin reduction assay. (**a**) Cell viability in MCF7, HCC38, MCF-10A, and MDA-MB-436 breast cancer cells treated with 1–10,000 nM bortezomib for 24 hours. Three independent experiments were performed in triplicate. Error bars depict the standard error of the mean. (**b**) Proteasome activity in MCF7, HCC38, MCF-10A, and MDA-MB-436 cells determined after two hours of bortezomib exposure. T-test was used to calculate statistical significance (Benjamini-Hochberg adjusted p-values). ns = not significant (P > 0.05); *P ≤ 0.05; **P ≤ 0.01; ***P ≤ 0.001; ****P ≤ 0.0001. (**c**) Bortezomib induces G2/M arrest in HCC38 cells treated with 5 nM bortezomib for 24 hours. The dose-response curves and bar plots were generated using ggplot2 (version 3.2.1) in R^51^. The cell cycle distribution graphs were generated using the Flowing Software (http://flowingsoftware.btk.fi/index.php?page=1).

The 50% inhibitory concentration (IC50) is conventionally used to determine drug potency with cell-based cytotoxicity tests. According to the IC50 values, HCC38 cells were more sensitive to bortezomib than MCF7, MCF-10A, and MDA-MB-436 cells 24 hours after treatment (Fig. 6a). In line with Hafner *et al*., IC50 values were shown to be dependent on cell growth rates and drug treatment time^6^. For the HCC38 and MCF7 cell lines, IC50 values were found to decrease with increasing bortezomib treatment times and resulted in relatively similar IC50 values 72 hours post-treatment (Supplementary data; MCF7, IC50 = 37 nM and HCC38, IC50 = 2.5 nM). These findings prompted us to investigate whether bortezomib was an effective proteasome inhibitor. At least 50% inhibition of proteasome activity was detected in all four cell lines two hours after exposure to 10 nM bortezomib and close to complete proteasome inhibition with 100 nM bortezomib (Fig. 6b). As expected, bortezomib blocked cell cycle progression by inducing G2/M phase arrest in HCC38 cells 24 h after treatment (Fig. 6c).

**Figure 7:**
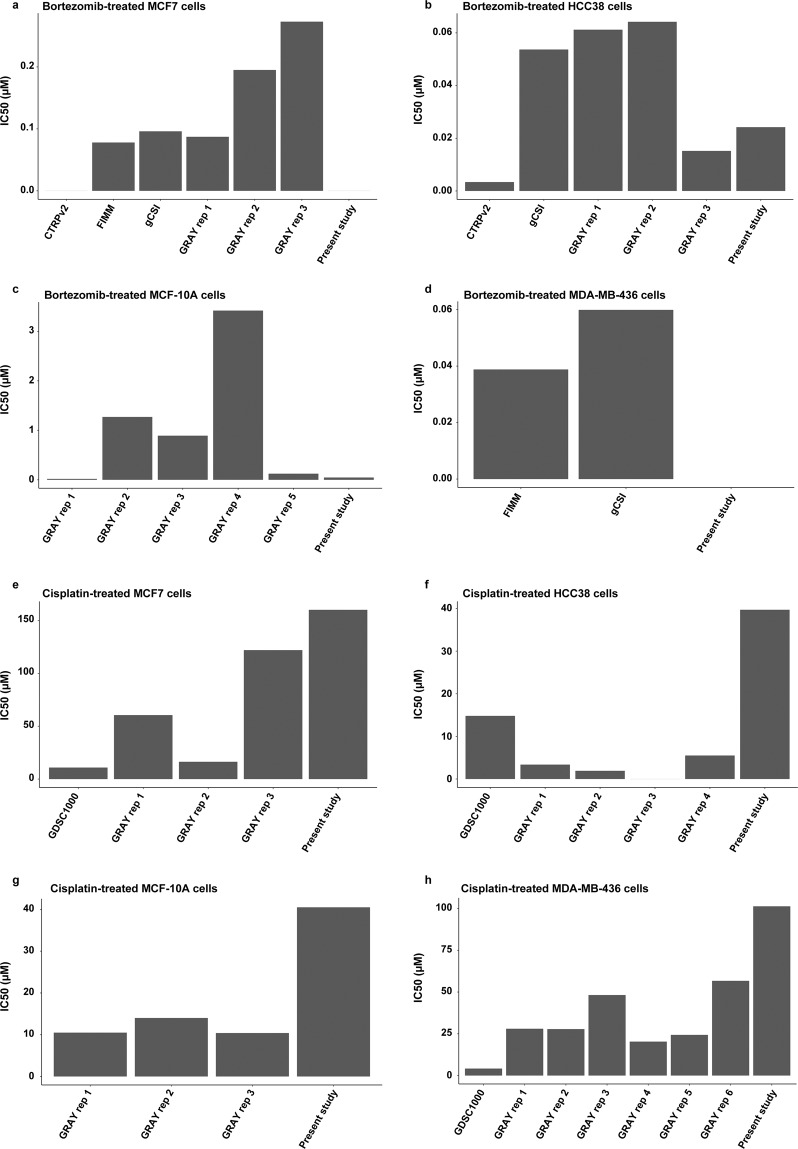
IC50 values for (**a–d**) bortezomib- and (**e–h**) cisplatin-treated MCF7, HCC38, MCF-10A, and MDA-MB-436 breast cancer cells generated in the present study and pharmacoDB^42^ datasets. No bar is shown for treated cells that do not reach the half maximal inhibitory concentration. The bar plots were generated using ggplot2 (version 3.2.1) in R^51^.

The pharmacoDB database was then used to compare the IC50 values for MCF7, HCC38, MCF-10A, and MDA-MB-436 cells treated with bortezomib and cisplatin (Fig. 7). On average, the pharmacoDB datasets showed IC50 values of 0.15 µM (range, from IC50 value not reached to 0.27 µM), 0.04 µM (range, 0.0034–0.064 µM), 1.1 µM (range, 0.017–3.4 µM), and 0.049 µM (range, 0.039–0.060 µM) for MCF7, HCC38, MCF-10A, and MDA-MB-436 cells treated with bortezomib, respectively. On the other hand, MCF7, HCC38, MCF-10A, and MDA-MB-436 treated with cisplatin showed IC50 values of 52 µM (range, 10–122 µM), 6.4 µM (range, from IC50 value not reach to 15 µM), 12 µM (range, 10–14 µM), and 30 µM (range, 4.1–57 µM), respectively. Although IC50 values were generally within range for bortezomib for all cell lines in the present study, our data showed slightly higher IC50 values for cisplatin.

**Figure 8:**
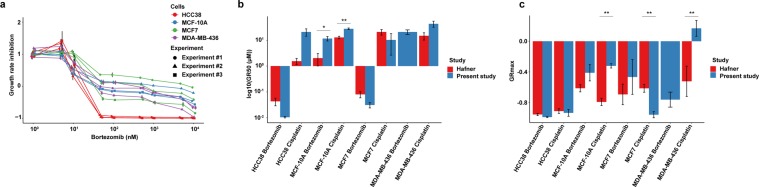
Growth rate inhibition data generated after optimization of the resazurin reduction assay. (**a**) Drug potency and efficiency was determined on MCF7, HCC38, MCF-10A, and MDA-MB-436 cell lines using growth rate inhibition metrics (GR50 and GRmax). Three independent experiments were performed in triplicate. Error bars depict the standard error of the mean. (**b**) Comparison of GR50 and (**c**) GRmax values for bortezomib- and cisplatin-treated MCF7, HCC38, MCF-10A, and MDA-MB-436 breast cancer cells generated in the present study and Hafner *et al*.^31^ Error bars depict the standard error of the mean. T-test was used to calculate statistical significance (Benjamini-Hochberg adjusted p-values). ns = not significant (P > 0.05); *P ≤ 0.05; **P ≤ 0.01; ***P ≤ 0.001; ****P ≤ 0.0001. The dose-response curves and bar plots were generated using ggplot2 (version 3.2.1) in R^51^.

On average, the estimated population doubling time for MCF-10A, MCF7, MDA-MB-436, and HCC38 cells was 21, 26, 35, and 36 hours. The MCF7 doubling time (26 hours) was in agreement with those determined in the NCI-60 cancer cell line panel (26 hours)^30^. Due to the difference in population doubling times, growth inhibition metrics (GR50, GRmax, and GR_AOC_) were used to assess drug potency and efficiency after adjusting for cell growth rates. Using these metrics, we found that cisplatin was comparably cytotoxic to HCC38 (GR50 = 21 ± 7 µM, GRmax = −0.93 ± 0.05, and GR_AOC_ = 1.2 ± 0.1), MCF7 (GR50 = 15 ± 10 µM, GRmax = −0.96 ± 0.04, and GR_AOC_ = 1.0 ± 0.2), and MDA-MB-436 (GR50 = 9.4 ± 1.7 µM, GRmax = −0.90 ± 0.07, and GR_AOC_ = 1.1 ± 0.1) than MCF-10A cells (GR50 = 29 ± 4 µM, GRmax = −0.69 ± 0.09, and GR_AOC_ = 0.9 ± 0.1). In addition, HCC38 cells (GR50 = 10 ± 1 nM; GRmax = −0.99 ± 0.01, and GR_AOC_ = 1.3 ± 0.0) were shown to be more sensitive to bortezomib than the other three cell lines (MCF7 cells, GR50 = 31 ± 7 nM, GRmax = −0.43 ± 0.23, and GR_AOC_ = 0.72 ± 0.14; MDA-MB-436, GR50 = 21 ± 4 nM, GRmax = −0.76 ± 0.10, and GR_AOC_ = 0.85 ± 0.05; MCF-10A, GR50 = 12 ± 2 nM, GRmax = −0.41 ± 0.11, and GR_AOC_ = 0.82 ± 0.06), with differing GRmax values indicating variable cytotoxic response to bortezomib (Fig. 8a). Interestingly, in agreement with Hafner *et al*. HCC38 cells were shown to be more sensitive to bortezomib than MCF7 and MCF-10A cells, and GRmax values revealed that bortezomib and cisplatin were cytotoxic to all cell lines except for MDA-MB-436^31^. However, Hafner’s data indicated that HCC38 cells were more sensitive to cisplatin than MCF7 cells, whereas our data showed no difference in sensitivity to cisplatin. In addition, we found a statistically significant difference in GR50 values for our two studies for MCF-10A cells treated with bortezomib and cisplatin, as well as, GRmax values for cisplatin-treated MCF-10A, MCF7, and MDA-MB-436 cells (Fig. 8b,c).

## Discussion

In the current study, we describe a number of biological (cell type, medium composition and volume, and seeding density) and technical (edge effect, drug type, dose, storage, and treatment time, assay duration time, dose-response metric) factors that should be taken into consideration to achieve more reliable and reproducible drug-dose sensitivity screens in 2D models. Although many of these factors are known to affect drug response, few studies have proposed strategies to quantify and correct sources of experimental variability in cell-based drug screens using QCM^6–8,16,18,19,32^. The optimization strategy used here can be adapted to other cell-based and animal model systems by first identifying confounding factors in the experimental setup, followed by optimization of critical experimental parameters and assessment of data quality. However, it is recommended that each lab evaluates whether the proposed optimal conditions are suitable for the cells (*e.g*. derived from other tissue types) and assay (*e.g*. signal dynamic range) used in their experiments. Here, we first optimized the resazurin viability assay using two breast cancer cell lines (HCC38 and MCF7) and then implemented the optimized assay on two additional cell lines derived from breast tissue (MCF-10A and MDA-MB-436). We used the resazurin substrate as a marker of cell viability because it is considered to be a relatively quick, non-toxic, cost-effective, and flexible method that can be utilized with a variety of different culture conditions^8,33,34^. To be able to compare drug potency estimates between different studies, long-established breast cancer cell lines (HCC38, MCF7, MCF-10A, and MDA-MB-436) and pharmaceutical agents (bortezomib, cisplatin, and carboplatin) in cancer research and drug screening were utilized^35–37^.

There has been considerable controversy whether high-throughput screens (HTS) produce consistent results between different centers^7,13^. Despite differences in experimental protocols, two large-scale HTS pharmacogenomics studies (Cancer Genome Project (CGP) and the Cancer Cell Line Encyclopedia (CCLE)) were shown to generate highly concordant transcriptional profiles, but discordant drug response. These findings indicate a need to standardize protocols for cell-based drug sensitivity screens by *e.g*. establishing a consensus on appropriate positive controls (genomically verified cancer cell lines treated with “reference” chemotherapeutic agents), cell culture conditions, etc^8^. To address this issue, use of long-established breast cancer cell lines and pharmaceutical agents were used in the present study. However, HTS are frequently performed using automated liquid handling robots. Although robotic systems are time effective and can facilitate high precision pipetting, challenges associated with this method include *e.g*. the inability to adjust drug doses for different cell types, evaporation (edge effect), clogging, air bubbles, use of small volumes and pipetting of viscous liquids^38^. In order to develop common standards for drug screening, it is also important to investigate the effect of confounding factors on cell viability and identify drugs that have a cytotoxic effect by evaluating assay suitability with QCM^28^.

Drug screening assays with a low CV (little to no signal variation), high SW (above 2 SD, large signal dynamic range) and a Z-factor close to or equal to 1 should be able to identify biologically active pharmaceutical compounds. Here, CV, SW, and Z-factor were within recommended ranges (CV < 20%, SW > 2 SD, and Z-factor > 0.4) in the three independent experiments, with CV below 5%, SW exceeding 10 SD and Z-factor close to 1. Furthermore, variance component analysis can potentially identify confounders in an assay by estimating the contribution of covariates (*e.g*. cell type, drug, drug dose) to the variance of a dependent variable (*e.g*. cell viability). In agreement with previous studies, we demonstrated that cell line, drug type, dose and treatment time had a substantial effect on cell viability^8,16^. Though we used cell lines derived from different breast cancer subtypes with differing biological features, the impact of cell type on drug response was likely underestimated due to the use of cell lines from the same tissue. Seeding density, medium type and volume, and resazurin assay time were also shown to have an impact on cell viability, though to a lesser extent^6,8^. In the present study, a narrow range of seeding densities (5000 to 10000 cells/well) was used. However, Hafner *et al*. showed that seeding density can have a major effect on GR values, especially when using a broad range of seeding numbers^6^. In contrast, the effect of medium type and volume is less studied. Interestingly, we show that different FBS concentrations (0–20% FBS) have a minimal effect on drug sensitivity after 24 hours exposure. As FBS is expected to have an effect on cell proliferation, it would be interesting to assess the impact of FBS concentration on cell viability over time^39,40^.

Interexperimental variability was reduced by optimizing critical experimental parameters (*e.g*. controls, edge effect, evaporation, and cell seeding density) for each cell line, thereby improving replicability of the drug-dose response curves. Pharmaceutical drugs are typically dissolved in various solvents (*e.g*. DMSO, DMF, saline, PBS) for use in *in vitro* studies. However, solvents such as DMSO can have a profound effect on cell viability, even at concentrations as low as 0.33%. By using a single DMSO vehicle control, we not only observed both over- and underestimation of viability, but also dose-response curves starting at levels above 100% viability. In contrast, matched DMSO concentration controls are highly recommended as they reduce the risk of dose curves starting at >100% viability. Due to the risk of evaporation, matched controls and drugs should be plated in the same location on the 96-well plate. Subsequent use of matched concentration controls is more time consuming because *e.g*. a dilution series of the solvent needs to be prepared, which takes up more space on the 96-well plate and drives up costs. In the edge effect experiments, we concluded that evaporation was higher around the perimeter of the 96-well plates, thereby affecting the concentration of added components and cell viability measurements. To minimize the edge effect, we excluded perimeter wells and instead filled these wells with PBS. Since the cell viability assay is usually performed over several days, each 96-well should be seeded with an appropriate cell number so that the cell population will be in the exponential phase during the course of the experiment and not affected by high confluency (stationary phase) that in turn could affect drug efficiency^31,41^. Lastly, an evaluation of evaporation on plates containing diluted drugs (stored at −20 °C) demonstrated that 96-well flat-bottom plates sealed with parafilm had higher evaporation than 96-well PCR plates sealed with aluminum tape. Consequently, evaporation of diluted drugs and medium components can lead to fluctuations in drug response, thereby making it difficult to estimate accurate IC50, GR50 and GRmax values and achieve reproducible results.

To evaluate the reproducibility of our results, we compared our data for four breast cancer cell lines (HCC38, MCF7, MCF-10A, and MDA-MB-436) treated with two anti-cancer drugs (bortezomib and cisplatin) with data from pharmacoDB and Hafner *et al*.^31,42^. Although we frequently report slightly higher IC50 values due to differences in drug treatment time (24 hours vs 48–72 hours), drug response was relatively comparable. With the exception of MCF-10A cells (treated with bortezomib and cisplatin) and MCF7/MDA-MB-436 cells treated with cisplatin, we show similar GR values as Hafner and colleagues. Drug treatment time differs frequently between studies, ranging from minutes to days. Here, we conducted drug sensitivity screening for 24, 48, 72 hours and observed similar IC50 values between different cell lines after 48 hours despite differences in population doubling time. In addition, medium and drug replacement every 24 hours had no effect on cell viability, indicating that treatment was most crucial during the first 24 hours. In the case of bortezomib, proteasome activity was inhibited after a 2 hour treatment time and G2/M phase arrest was induced within 24 hours. Consequently, the half-life of the tested drugs ranges from 25 minutes to about 20–30 hours in patients (depending on the drug type)^36,43,44^.

In summary, optimization is crucial for data reliability regardless of the model type (*e.g*. 2D monolayers, 3D spheroids or *in vivo* models) used to evaluate drug sensitivity^45^. We identified new critical experimental parameters (*e.g*. matched solvent concentration controls and drug storage) that need to be optimized to develop high precision, robust and reproducible cell viability assays. IC50 is commonly used by researchers to determine the potency of a drug on a certain cell line. To be able to compare drug potency estimates between different studies, we recommend using both IC50 and GR50. Human error or instrument inconsistencies can result in variations in data measurements that need to be overcome to identify biologically active hits (potent drugs)^28^. However, using QCM metrics increases the chance to identify hits within experiments. Ultimately, careful consideration to assay optimization and estimation of drug potency during the preclinical drug screening process may help to improve the success rates of cancer drug candidates that reach clinical trials.

## Methods

### Culture conditions

Human breast cancer cell lines (HCC38, MCF7, MCF-10A and MDA-MB-436) were purchased from the American Type Culture Collection (ATCC; Rockville, MD, USA) and cultured at 37 °C in a humidified 5% CO_2_ environment in RPMI 1640 (HCC38) supplemented with 2 mM L-glutamine, 2 g/L D-glucose, and 10% fetal bovine serum (FBS; Gibco ThermoFisher); RPMI 1640 (MCF-10A) supplemented with 2 mM L-glutamine, 2 g/L D-glucose, hydrocortisone, epidermal growth factor, cholera toxin, insulin, and 10% fetal bovine serum (FBS; Gibco ThermoFisher) or Dulbecco Modified Eagle’s Medium (DMEM; MCF7 and MDA-MB-436) supplemented with 2 mM L-glutamine, 4 g/L D-glucose, and 10% FBS (Gibco ThermoFisher). Cell growth rates were determined by non-linear regression curve analysis. Authentication of each cell line was performed using the ATCC short tandem repeat (STR) profiling service.

### Pharmaceutical compounds

Stock solutions for platinum-based agents (10 mM cisplatin and carboplatin) and a proteasome inhibitor (1 mM bortezomib) were prepared using DMSO (Sigma-Aldrich; stored at −80 °C), further diluted in 1xPBS to the appropriate concentration, and plated in 96-well PCR plates (VWR; stored at −20 °C). The pharmaceutical compounds were screened at nine concentrations (2–1024 µM cisplatin/carboplatin and 1–10,000 nM bortezomib) using a 2-fold dilution series with matched DMSO concentration vehicle controls. The pharmaceutical compounds were at room temperature (18-25 °C) when added to cells. Proteasome activity was assessed using the Proteasome-Glo Chymotrypsin-like assay (Promega) with bortezomib-treated cells seeded in 96-well clear, flat-bottom microplates (Corning Life Sciences) at a density of 7.5 × 10^3^ cells per well in 100 µl culture medium (RPMI or DMEM basal medium supplemented with 5%, 10% or 15% FBS or without FBS, and HuMEC Basal Serum-Free medium supplemented with epidermal growth factor, hydrocortisone, isoproterenol, transferrin, insulin, and bovine pituitary extract (Life Technologies)).

### Resazurin-based cell viability assay

Cells were plated in 96-well clear, flat-bottom microplates (Corning Life Sciences), at a density of 7.5 × 10^3^ cells per well in 100 µl culture medium supplemented with 10% FBS and cultured for 24 hours. Cell viability was assessed after drug treatment for 24, 48 or 72 hours using 0.2 mg/ml resazurin solutions prepared from resazurin sodium salt (Fisher Scientific) dissolved in sterile 1xPBS (ThermoFisher Scientific). In brief, resazurin was at room temperature before adding to the cells in each well. The cells were incubated with 10 µl resazurin solution (10% of cell culture volume) for four hours at 37 °C. The absorbance was measured with a 560 nm excitation filter and a 615 nm emission filter in a Wallac 1420 VICTOR2 microplate reader (Perkin Elmer). For five days, the metabolic activity of each cell line (untreated cells) was measured daily to monitor potential resorufin (fluorescent pink) conversion to dihydroresorufin (non-fluorescent and colorless). Optimization of the resazurin assay parameters is detailed in the Supplementary data.

Percentage cell viability was calculated as 100% × (absorbance of treated cells – absorbance of background controls) / (absorbance of matched DMSO concentration controls – absorbance of background controls). Normalized growth rate inhibition (GR) was assessed using the absorbance of untreated cells at the time of treatment (t = 0; 24 hours after plating). The half-maximal inhibitory concentration (IC50), drug potency (GR50), and drug efficiency (GRmax) were determined for each compound using the GRmetrics (version 1.0.0) package^46^ in R/Bioconductor (version 3.3.2).

### Cell population doubling time

HCC38 and MCF7 cells were plated in 96-well flat-bottom plates (Corning Life Science) at a density of 7.5 × 10^3^ cells per well in 100 µl culture medium supplemented with 10% FBS and cultured for five days. The resazurin assay was performed daily to determine the cell population doubling time (T*d*) during the exponential growth phase. The doubling time was calculated in hours using linear regression analysis.

### Flow cytometry-based cell cycle distribution analysis

HCC38 cells were cultured in 10-cm dishes with either different drug concentrations or DMSO (vehicle control) for 24 hours. Cell cycle distribution analyses were performed using harvested cells, fixed with 70% ethanol and stained with propidium iodide/RNase staining solution (Cell Signaling Technology). Apoptosis was assessed using the sub-G1 cell cycle fraction. Data analysis for cell cycle distribution was performed using the FACScalibur system (BD Biosciences) and Flowing software (version 2.5.1).

### Statistical analysis

A variance component analysis was used to determine the influence of experimental factors (*e.g*. cell line, drug, drug dose) in the resazurin-based cell viability assay, analysis of variance (ANOVA), Z-factor (Z), signal window (SW), and coefficient of variation (CV) were calculated as described elsewhere^27,47^, with cutoffs set at Z > 0.4, SW > 2, and CV < 20%. In brief, one-way ANOVA (base stats package in R/Bioconductor version 3.3.2) was performed using cell viability as the dependent variable and all other variables (*e.g*. cell line, drug, drug dose) as covariates. The ANOVA percentage of variation explained (η²) was calculated by dividing the sum of squares (Sum Sq) between groups by the Total Sum Sq (Supplementary Table 1). The Z, SW, and CV scores were calculated using resazurin-based absorbance values for treated cells and background controls, as previously described^47^. To evaluate edge effect, i.e. differences in resazurin-based absorbance values in edge and middle wells on a 96-well plate, Wilcoxon rank-sum test (*P* < 0.05) was used. The Shapiro-Wilk normality test was used in R to determine whether the data was normally distributed, where the parametric T-test was used if *P* > 0.05 (normally distributed) or the non-parametric Wilcoxon test was used if *P* < 0.05 (not normally distributed). Bar plots were constructed using the ggpubr (version 0.2.1.999)^48^ and rstatix (version 0.1.1.999)^49^ R packages to compare different groups with T-test or Wilcoxon test and Benjamini-Hochberg adjusted p-values, as appropriate.

## Supplementary information

Supplementary Information.

Supplementary Table S2

## Data Availability

The dataset and source codes for this work are publicly available as a capsule on CodeOcean^50^.
