# Supplementary material for: Optimization of cell viability assays to improve replicability and reproducibility of cancer drug sensitivity screens
Source: Sci Rep. 2020 Apr 2;10:5798. doi: 10.1038/s41598-020-62848-5 (PMC7118156; doi:10.1038/s41598-020-62848-5)
Supplement: Supplementary file 1 — Supplementary Information. [file 41598_2020_62848_MOESM1_ESM.pdf]

Optimization of cell viability assays to improve replicability and reproducibility of cancer drug sensitivity screens

Peter Larsson, Hanna Engqvist, Jana Biermann, Elisabeth Werner Rönnerman, Eva Forssell-Aronsson, Anikó Kovács, Per Karlsson, Khalil Helou, and Toshima Z. Parris

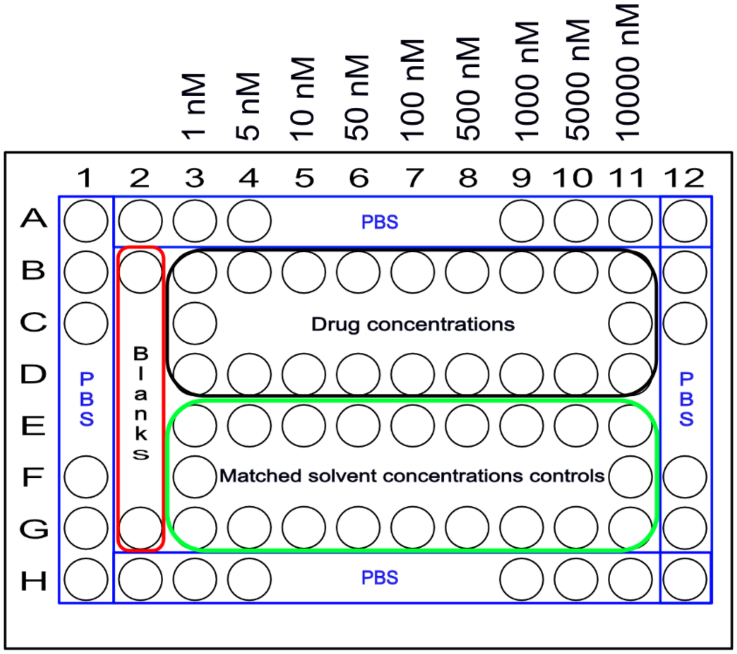

Supplementary Figure 1. Sample plating setup. Every tested concentration was performed in triplicates. BLANK contains only medium. Matched DMSO concentration controls used to exclude the DMSO toxicity. PBS around the edges to minimize edge effect.

Supplementary data

Assay optimization

Evaporation in 96-well microplates

Evaporation in 96-well flat-bottom culture microplates stored at 4°C and -20°C

HCC38 cells were seeded in 96-well flat-bottom microplates at a density of $1.0x10^4$ per well in 100 µl growth medium supplemented with 10% FBS and incubated for 24 hours at 37°C. A working solution of carboplatin was stored at 4°C or -20°C for 48 hours in 96-well flat-bottom culture microplates sealed with Parafilm around the lid. On day 1, cells were treated with freshly prepared carboplatin solutions. After 48 and 72 hours, the drug solutions stored in the freezer were thawed prior to drug treatment. The cells were treated with 4-1024 µM carboplatin for 24 hours and cell viability determined after incubation with 10% resazurin working solution for 4 hours (Supplementary Data file 1).

Evaporation in different types of 96-well microplates stored at -20°C

A working solution containing 16 nM bortezomib (200 µl per well) was plated in standard TC-treated 96-well flat-bottom (Corning Life Sciences) and PCR plates (VWR). The PCR plate was sealed with aluminum PCR sealing film and the flat-bottom plate with Parafilm around the plastic lid. After 48 and 72 hours, the flat-bottom plate was thawed and the volume measured with a manual pipette in randomly chosen wells. The PCR plate was thawed and quickly centrifuged before removing the tape and measuring the volume in the same wells as the flat-bottom plate. An assessment of signal variability was performed to compare the degree of liquid evaporation between the two 96-well plates.

Evaporation in 96-well flat-bottom microplates stored in a humidified 5% CO_2 environment

HCC38 cells were seeded in 96-well flat-bottom microplates at a density of 7.5×10^3 per well in 100 µl growth medium supplemented with 10% FBS and incubated for 24 hours at 37°C. Half of the cells on the 96-well plate were treated with 16 nM bortezomib and the other half with DMSO. Three randomly chosen outer/inner wells containing bortezomib or DMSO were measured with a manual pipette on day 1, day 2, and day 3. An assessment of signal variability was performed to compare the degree of liquid evaporation between the different wells.

Edge effect in 96-well flat-bottom microplates stored in a humidified 5% CO_2 environment

HCC38 cells were seeded in four 96-well flat-bottom microplates at a density of 7.5×10^3 per well in 100 µl growth medium supplemented with 10% FBS and incubated for 24 hours at 37°C. The cells were treated for 24 hours at 37°C with a) a full 96-well plate containing 16 nM bortezomib, b) a 96-well plate containing 16 nM bortezomib with 1xPBS in the outer wells, c) a full 96-well plate containing DMSO (same DMSO concentration used for bortezomib), and d) 96-well plate containing DMSO with 1xPBS in the outer wells. After 24 hours, cell viability was determined following incubation with 10% resazurin working solution for 4 hours.

Cells

Seeding density

HCC38 and MCF7 cells were seeded in 96-well flat-bottom microplates at densities of 5.0×10^3, 7.5×10^3, and 1.0×10^4 cells per well in 100 µl cell growth medium supplemented with 10% FBS and incubated for 24 hours at 37°C. The cells were treated with carboplatin or cisplatin for 24 hours and cell viability determined after incubation with 10% resazurin working solution for 4 hours (Supplementary Data file 2).

Sensitivity to drug solvent (DMSO)

MCF7 cells were seeded in 96-well flat-bottom microplates at a density of 7.5×10^3 per well in 100 µl growth medium supplemented with 10% FBS and incubated for 24 hours at 37°C. A 96-well PCR plate was prepared with a dilution series containing 1xPBS and DMSO diluted in 1xPBS (0.33-30% (v/v) DMSO). After 24 hours, cell viability was determined following incubation with 10% resazurin working solution for 4 hours (Supplementary Data file 3).

Medium

Type of growth medium

HCC38 cells were seeded in 96-well flat-bottom microplates at a density of 7.5×10^3 per well in 100 µl growth medium (RPMI, RPMI+5% FBS, RPMI+10% FBS, RPMI+15% FBS, and HuMEC Basal Serum-Free Medium supplemented with epidermal growth factor, hydrocortisone, isoproterenol, transferrin, insulin, and bovine pituitary extract (Life Technologies)). MCF7 cells were seeded in 96-well plates at a density of 7.5×10^3 per well in 100 µl growth medium (DMEM, DMEM+5% FBS, DMEM+10% FBS, DMEM+15% FBS, and supplemented HuMEC medium). HCC38 cells were treated for 24 hours with bortezomib, while MCF7 cells were treated with cisplatin. Cell viability was determined 24 hours post drug exposure after incubation with 10% resazurin working solution for 4 hours (Supplementary Data file 4).

Volume of growth medium per 96-well

HCC38 and MCF7 cells were seeded in 96-well flat-bottom microplates at a density of 7.5×10^3 per well in 50 µl (only for MCF7 cells), 100 µl, 200 µl, and 240 µl growth medium supplemented with 10% FBS and incubated for 24 hours at 37°C. The cells were treated with carboplatin or cisplatin for 24 hours and cell viability determined after incubation with 10% resazurin working solution for 4 hours (Supplementary Data file 5).

Long-term drug exposure and growth medium/drug renewal

HCC38 and MCF7 cells were seeded in 96-well flat-bottom microplates at a density of 7.5×10^3 per well in 100 µl growth medium supplemented with 10% FBS and incubated for 24 hours at 37°C. One batch of cells was treated with bortezomib for 24, 48, and 72 hours. Another batch of cells was treated with bortezomib for 24, 48, and 72 hours with cell culture medium and drug renewal every 24 hours. Cell viability was determined after incubation with 10% resazurin working solution for 4 hours (Supplementary Data file 6).

Effects of growth medium antibiotics

HCC38 and MCF7 cells were seeded in 96-well flat-bottom microplates at a density of 7.5×10^3 per well in 100 µl growth medium supplemented with 10% FBS and incubated for 24 hours at 37°C. Cells were cultured using growth medium supplemented with 1% penicillin-streptomycin (Life Technologies) or without penicillin-streptomycin (control group). HCC38 cells were treated for 24 hours with bortezomib, while MCF7 cells were treated with cisplatin. Cell viability was determined 24 hours post drug exposure after incubation with 10% resazurin working solution for 4 hours (Supplementary Data file 7).

Resazurin

Mode of resorufin detection

MCF7 cells were seeded in 96-well flat-bottom microplates at a density of 7.5×10^3 per well in 100 µl growth medium supplemented with 10% FBS, incubated for 24 hours at 37°C, and treated with bortezomib for 24 hours. Cell viability was determined 24 hours post drug exposure after incubation with 10% resazurin working solution for 4 hours. A Wallac 1420 VICTOR2 microplate reader (Perkin Elmer) was used to measure absorbance and fluorescence with a 560 nm excitation filter and a 615 nm emission filter.

Resazurin incubation time

HCC38 and MCF7 cells were seeded in 96-well flat-bottom microplates at a density of $7.5x10^3$ per well in 100 µl growth medium supplemented with 10% FBS and incubated for 24 hours at 37°C. HCC38 were treated for 24 hours with bortezomib, whereas MCF7 cells were treated with cisplatin. Cell viability was determined 24 hours post drug exposure with 10% resazurin working solution and different resazurin incubation time points (1, 2, 4 and 6 hours; Supplementary Data file 8).

Resazurin concentration

HCC38 and MCF7 cells were seeded in 96-well flat-bottom microplates at a density of $7.5x10^3$ per well in 100 µl growth medium supplemented with 10% FBS and incubated for 24 hours at 37°C. HCC38 and HCC1395 were treated for 24 hours with bortezomib, whereas MCF7 cells were treated with cisplatin. Cell viability was determined 24 hours post drug exposure after incubation with different concentrations of resazurin working solution (5%, 10%, 15% and 20%) for 4 hours (Supplementary Data file 9).

Cross-reactivity of resazurin with pharmaceutical compounds

Growth medium (RPMI and DMEM) supplemented with 10% FBS (100 µl) were added to 96-well flat-bottom microplates without cells and incubated with bortezomib, carboplatin or DMSO for 24 hours. The resazurin reduction assay was performed with 10% resazurin for 4 hours to assess the extent of resorufin production.

Evaporation due to storage at 4°C and -20°C

**Medium type:**	Growth medium + 10% FBS	**Treatment time:**	24h
**Medium volume/well:**	100 µl	**Controls:**	Matched DMSO concentration controls
**Seeding density:**	7.5x10^3 cells/well	**Resazurin incubation time:**	4h
**Drug(s):**	Carboplatin	**Resazurin concentration:**	10%
**Drug concentrations:**	2, 4, 8, 32, 64, 128, 256, 512, 1024 (µM)	**Blank:**	Medium only
**Drug storage:**	Drug stored at 4°C or -20°C for up to 48h and defrosted every 24h	**Plates:**	96-well flat-bottom plate

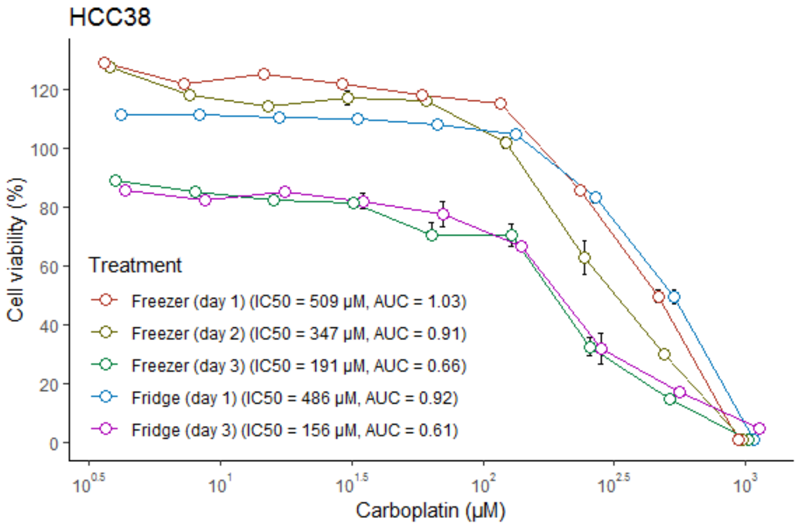

Supplementary Data file 1

Seeding density

**Medium type:**	Growth medium + 10% FBS	**Treatment time:**	24h
**Medium volume/well:**	100 µl	**Controls:**	Matched DMSO concentration controls
**Seeding density:**	$5.0x10^3$, $7.5x10^3$, $1.0x10^4$ cells/well	**Resazurin incubation time:**	4h
**Drug(s):**	Carboplatin, Cisplatin	**Resazurin concentration:**	10%
**Drug concentrations:**	2, 4, 8, 32, 64, 128, 256, 512, 1024 (µM)	**Blank:**	Medium only
**Drug storage:**	Drug stored at -20°C between experiments	**Plates:**	96-well flat-bottom plate

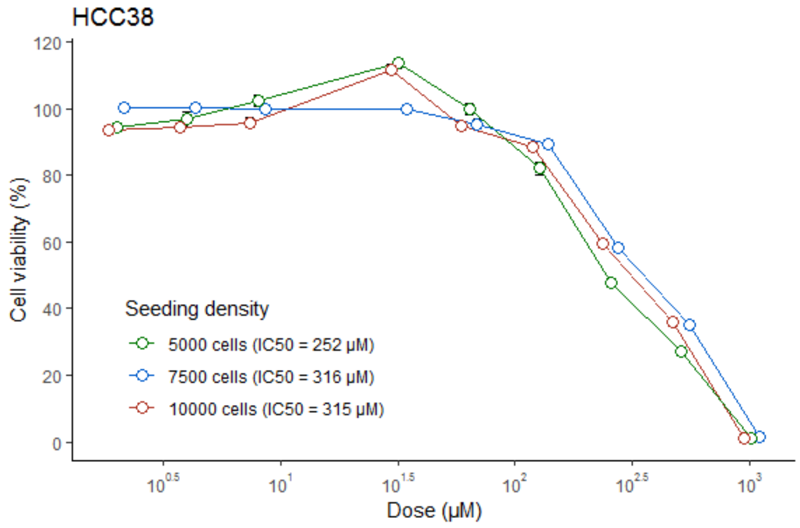

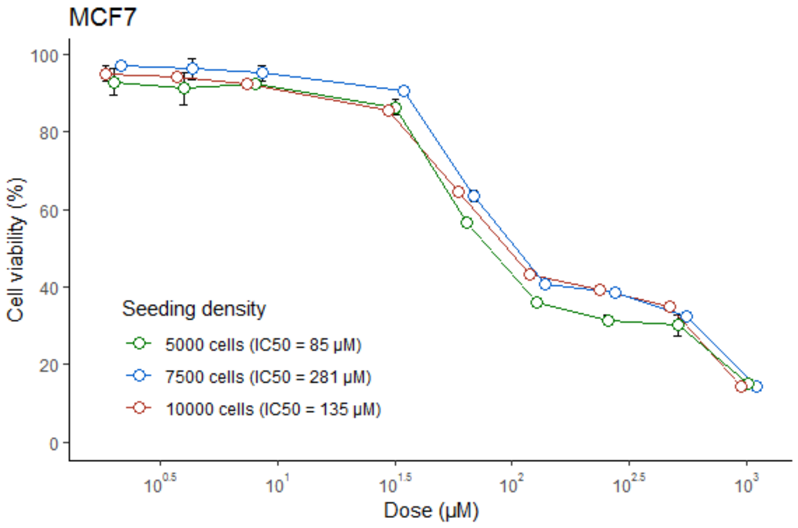

Supplementary Data file 2

Sensitivity to DMSO

**Medium type:**	Growth medium + 10% FBS	**Treatment time:**	24h
**Medium volume/well:**	100 µl	**Controls:**	Matched DMSO concentration controls
**Seeding density:**	7.5×10^3 cells/well	**Resazurin incubation time:**	1h, 2h, 4h, 6h
**Solvent:**	DMSO	**Resazurin concentration:**	10%
**Solvent concentrations:**	0.33, 0.5, 1, 2, 5, 10, 20, 30%	**Blank:**	Medium only
**Solvent storage:**	-	**Plates:**	96-well flat-bottom plate

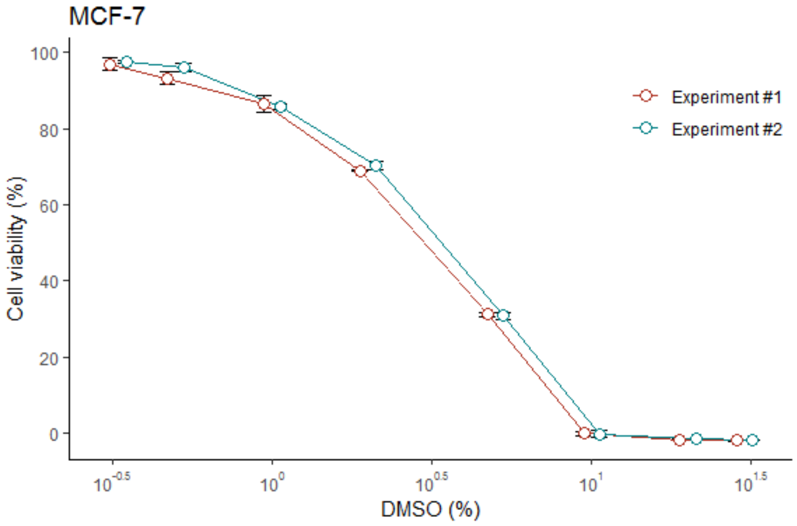

Supplementary Data file 3

Medium type

**Medium type:**	Growth medium + 5-15% FBS, serum-free HuMEC medium	**Treatment time:**	24h
**Medium volume/well:**	100 µl	**Controls:**	Matched DMSO concentration controls
**Seeding density:**	$7.5x10^3$ cells/well	**Resazurin incubation time:**	4h
**Drug(s):**	Bortezomib, Cisplatin	**Resazurin concentration:**	10%
**Drug concentrations:**	4, 20, 100, 500, 2500 (nM); 2, 4, 8, 32, 64, 128, 256, 512, 1024 (µM)	**Blank:**	Medium only
**Drug storage:**	Drug stored at -20°C between experiments	**Plates:**	96-well flat-bottom plate

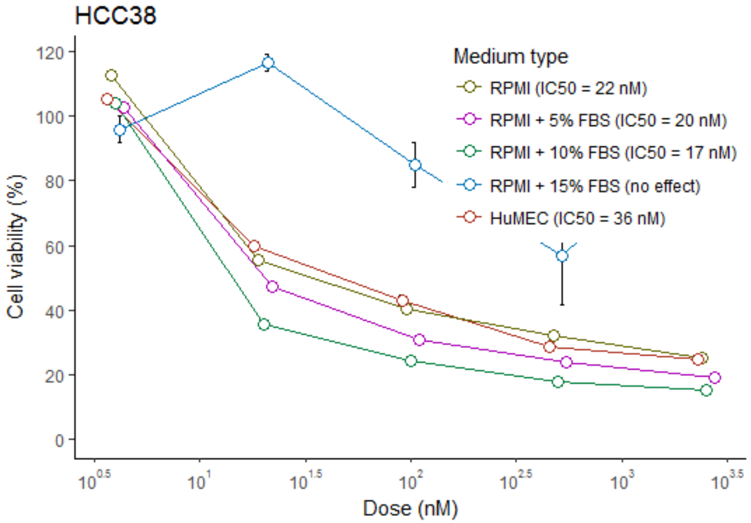

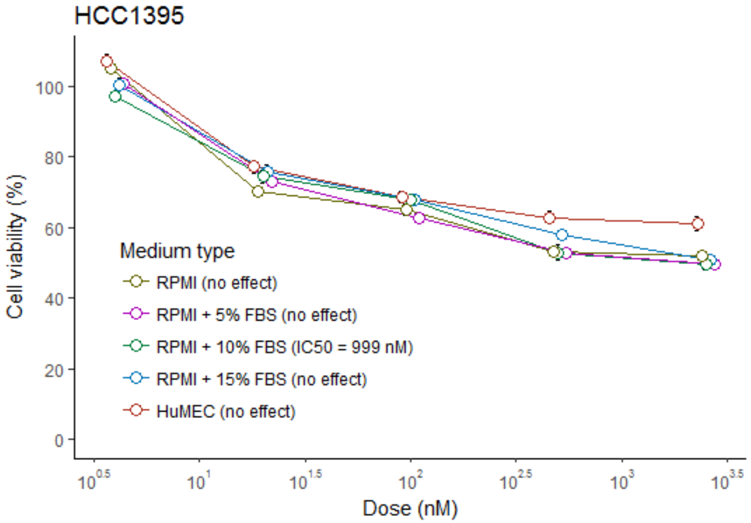

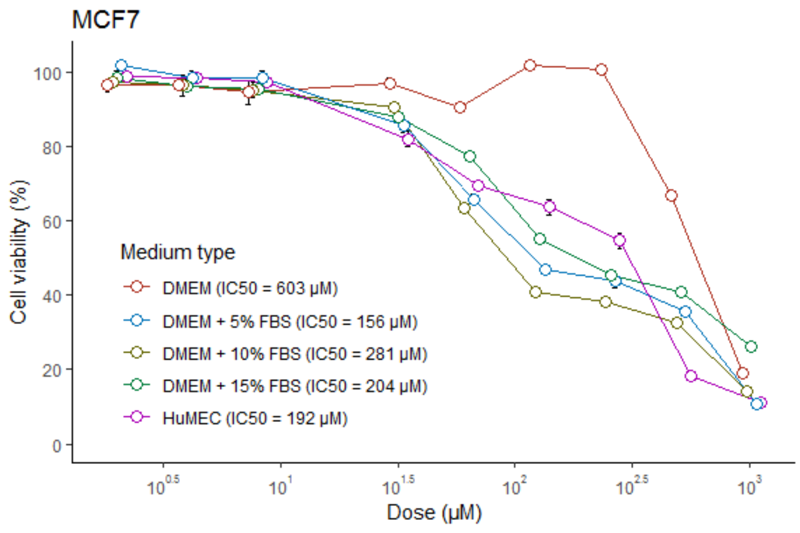

Supplementary Data file 4

Medium volume

**Medium type:**	Growth medium + 10% FBS	**Treatment time:**	24h
**Medium volume/well:**	100 µl, 200 µl, 240 µl	**Controls:**	Matched DMSO concentration controls
**Seeding density:**	7.5x10^3 cells/well	**Resazurin incubation time:**	4h
**Drug(s):**	Carboplatin, Cisplatin	**Resazurin concentration:**	10%
**Drug concentrations:**	2, 4, 8, 32, 64, 128, 256, 512, 1024 (µM)	**Blank:**	Medium only
**Drug storage:**	Drug stored at -20°C between experiments	**Plates:**	96-well flat-bottom plate

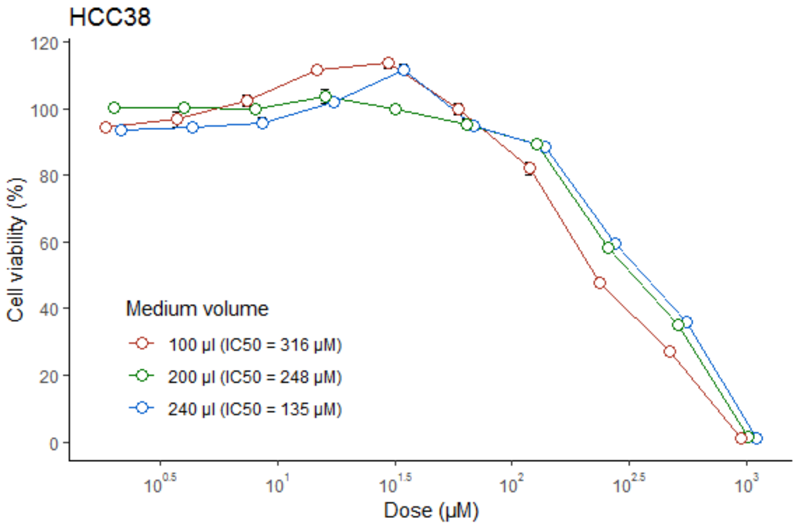

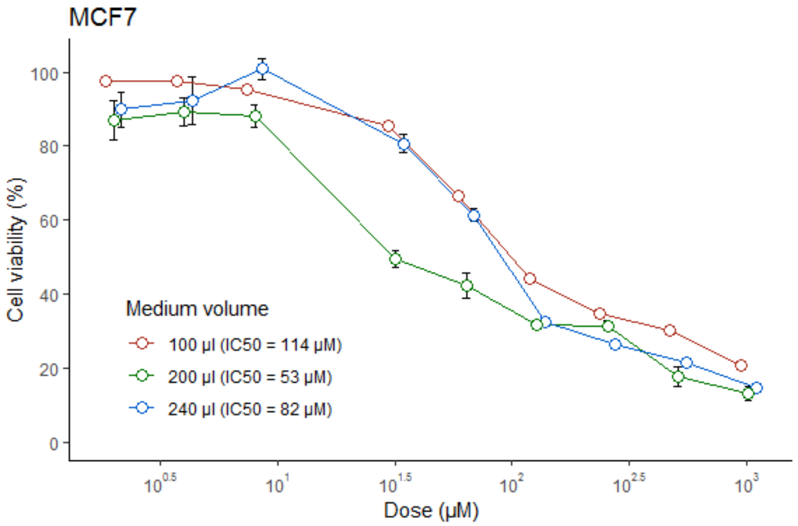

Supplementary Data file 5

Medium/drug renewal

**Medium type:**	Growth medium + 10% FBS	**Treatment time:**	24h, 48h, 72h (with or without medium/drug renewal every 24h)
**Medium volume/well:**	100 µl	**Controls:**	Matched DMSO concentration controls
**Seeding density:**	$7.5x10^3$ cells/well	**Resazurin incubation time:**	4h
**Drug(s):**	Bortezomib	**Resazurin concentration:**	10%
**Drug concentrations:**	1, 5, 10, 50, 100, 500, 1000, 5000, 10000 (nM)	**Blank:**	Medium only
**Drug storage:**	Drug stored at -20°C between experiments	**Plates:**	96-well flat-bottom plate

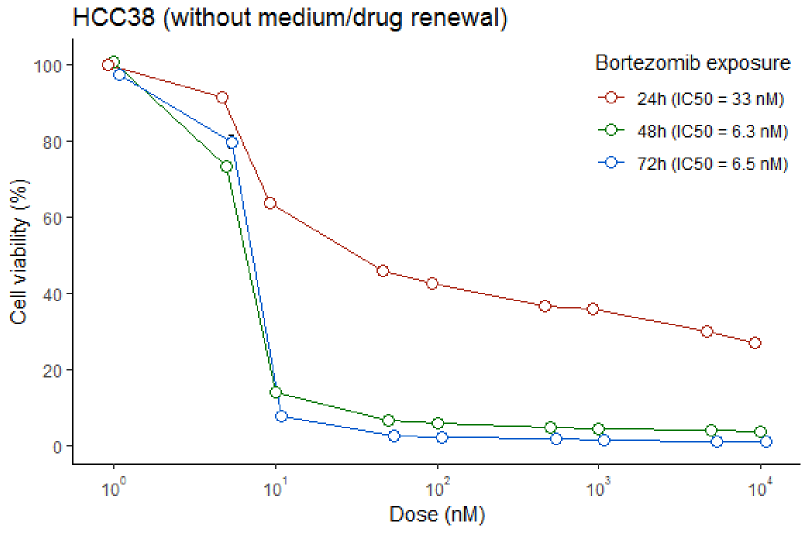

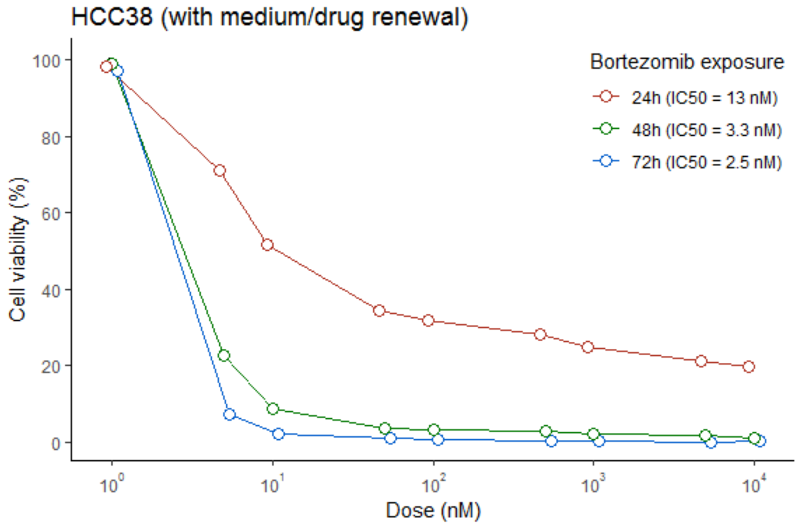

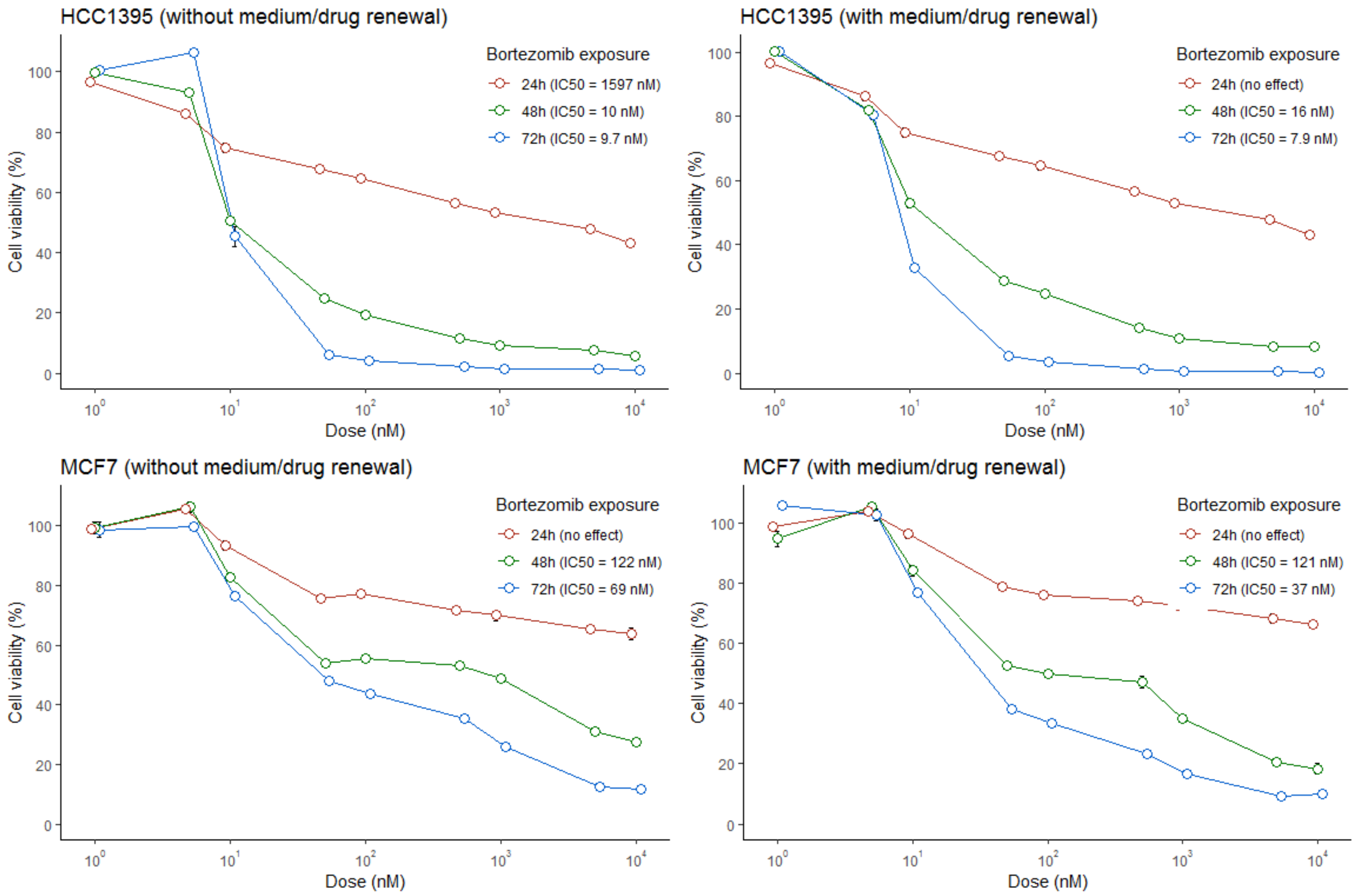

Supplementary Data file 6

Treatment with penicillin-streptomycin

**Medium type:**	Growth medium + 10% FBS (with or without penicillin-streptomycin)	**Treatment time:**	24h
**Medium volume/well:**	100 µl	**Controls:**	Matched DMSO concentration controls
**Seeding density:**	7.5×10^3 cells/well	**Resazurin incubation time:**	4h
**Drug(s):**	Bortezomib	**Resazurin concentration:**	10%
**Drug concentrations:**	1, 5, 10, 50, 100, 500, 1000, 5000, 10000 (nM)	**Blank:**	Medium only
**Drug storage:**	Drug stored at -20°C between experiments	**Plates:**	96-well flat-bottom plate

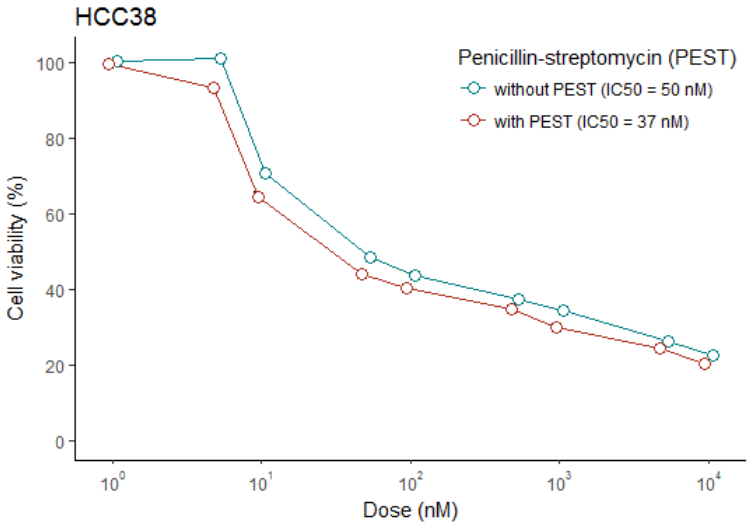

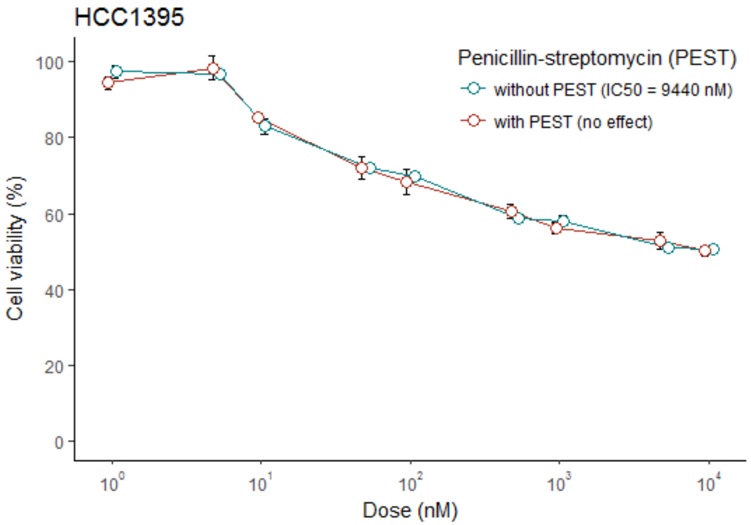

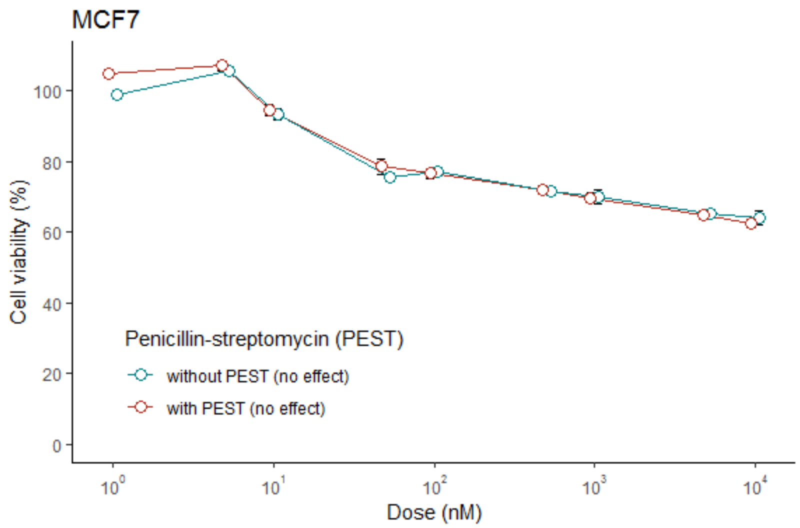

Supplementary Data file 7

Resazurin incubation time

**Medium type:**	Growth medium + 10% FBS	**Treatment time:**	24h
**Medium volume/well:**	100 µl	**Controls:**	Matched DMSO concentration controls
**Seeding density:**	7.5×10^3 cells/well	**Resazurin incubation time:**	1h, 2h, 4h, 6h
**Drug(s):**	Bortezomib, Cisplatin	**Resazurin concentration:**	10%
**Drug concentrations:**	1, 5, 10, 50, 100, 500, 1000, 5000, 10000 (nM); 2, 4, 8, 32, 64, 128, 256, 512, 1024 (µM)	**Blank:**	Medium only
**Drug storage:**	Drug stored at -20°C between experiments	**Plates:**	96-well flat-bottom plate

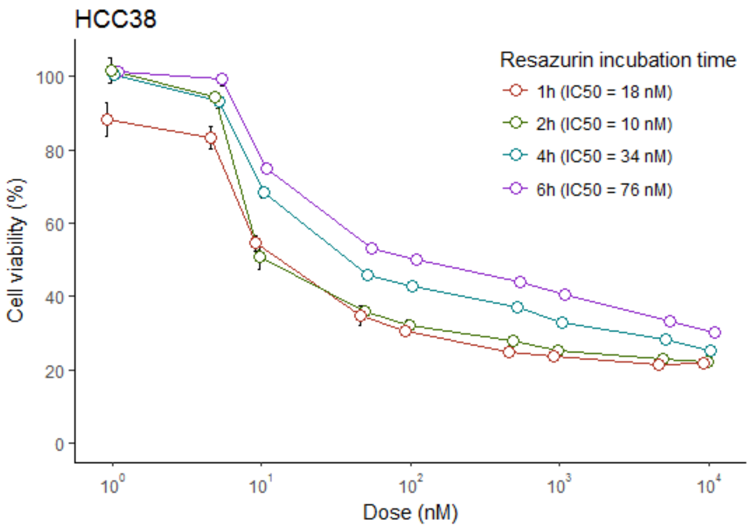

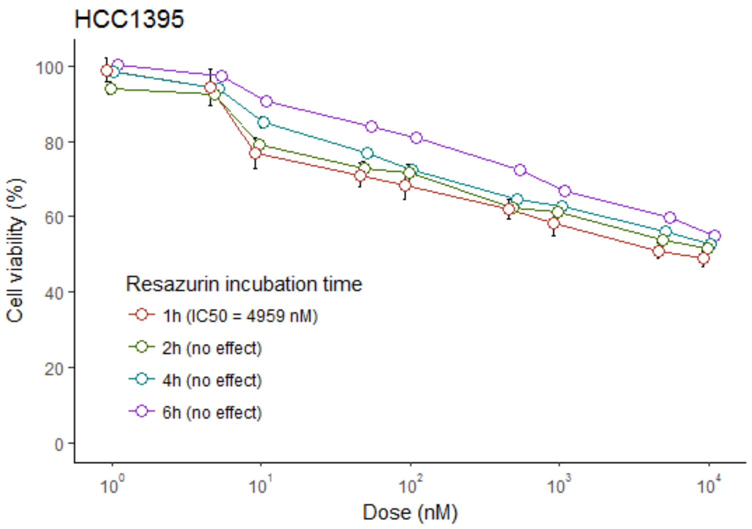

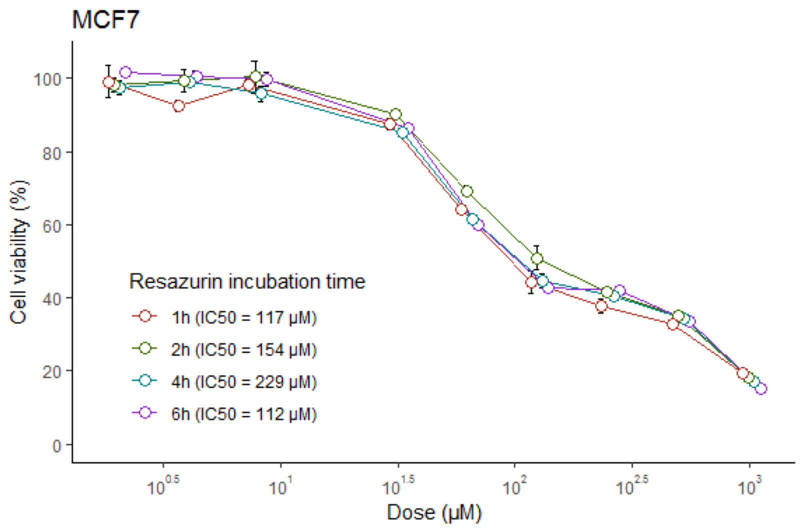

Supplementary Data file 8

Resazurin concentration

**Medium type:**	Growth medium + 10% FBS	**Treatment time:**	24h
**Medium volume/well:**	100 µl	**Controls:**	Matched DMSO concentration controls
**Seeding density:**	7.5x10^3 cells/well	**Resazurin incubation time:**	4h
**Drug(s):**	Bortezomib, Cisplatin	**Resazurin concentration:**	5-20%
**Drug concentrations:**	1, 5, 10, 50, 100, 500, 1000, 5000, 10000 (nM); 2, 4, 8, 32, 64, 128, 256, 512, 1024 (µM)	**Blank:**	Medium only
**Drug storage:**	Drug stored at -20°C between experiments	**Plates:**	96-well flat-bottom plate

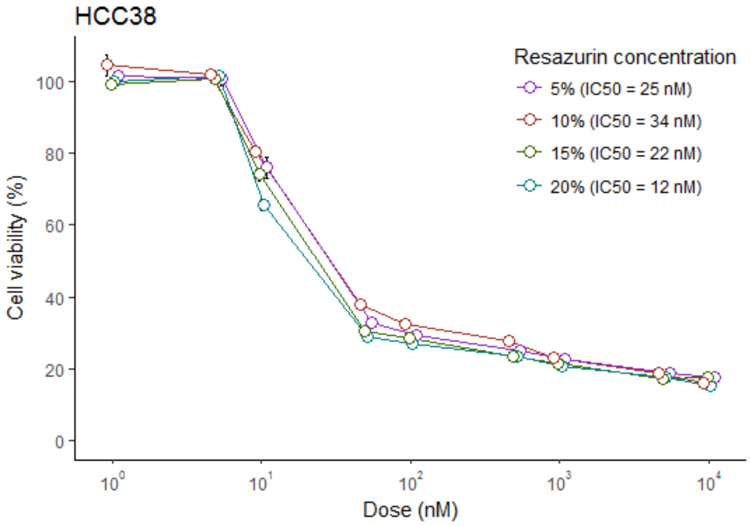

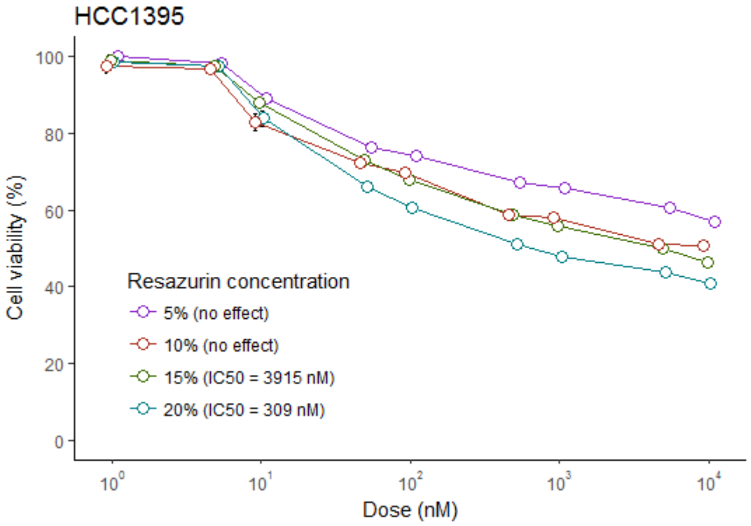

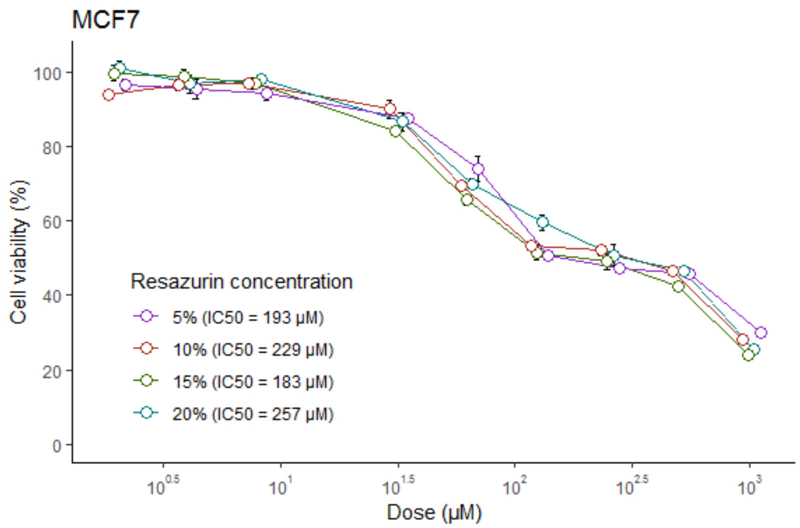

Supplementary Data file 9

Supplementary Table 1. Percentage of variance explained by experimental parameters

	Df	Sum Sq	Mean Sq	F value	Pr(>F)	% variance explained (η²)
Cell line	2	41385	20692	31.9	2.6E-14	5.4
Drug	2	162186	81093	124.9	2.8E-51	21.3
Drug dose	1	306958	306958	472.6	1.5E-92	40.3
Drug treatment time	2	221505	110753	170.5	3.7E-68	29.1
Medium type	4	8840	2210	3.4	8.8E-03	1.2
Medium volume	2	6474	3237	5.0	6.9E-03	0.8
Seeding density	1	117	117	0.2	6.7E-01	0.0
Resazurin concentration	3	2777	926	1.4	2.3E-01	0.4
Resazurin time	3	8167	2722	4.2	5.8E-03	1.1
Medium/drug renewal	1	2231	2231	3.4	6.4E-02	0.3
Antibiotics	1	1334	1334	2.1	1.5E-01	0.2
Residuals	1681	1091751	649.4649			
